# Effect of Dietary Addition of Blueberry (*Vaccinium corymbosum*) Powder on Fattening Performance, Meat Quality, Oxidative Stability and Storage Quality in Japanese Quails (*Coturnix coturnix japonica*)

**DOI:** 10.3390/ani15111633

**Published:** 2025-06-02

**Authors:** Shaistah Naimati, Sibel Canoğulları Doğan, Muhammad Umair Asghar, Qurat Ul Ain Sajid

**Affiliations:** 1 Department of Animal Production and Technologies, Faculty of Agricultural Sciences and Technologies, Niğde Ömer Halisdemir University, Niğde 51240, Türkiye; shaistah_naimati@mail.ohu.edu.tr (S.N.); scanogullari@ohu.edu.tr (S.C.D.); 2 Department of Animal Nutrition and Feed Sciences, Wroclaw University of Environmental and Life Sciences, 25 C.K. Norwida St., 51-630 Wrocław, Poland; qurat.ul.ain.sajid@upwr.edu.pl

**Keywords:** antioxidant, blueberry powder, Japanese quail, meat shelf life, performance

## Abstract

This study was conducted to investigate the effects of dietary addition of blueberry (*Vaccinium corymbosum*) powder on the growth performance, meat quality, oxidative stability and cold storage quality of Japanese quails (*Coturnix coturnix japonica*). In this research, 480 quail chicks were divided into four experimental groups, and each experimental group was composed of four replicates, each containing 30 quail chicks. Commercial feed was used in the study, but BBP was added to the feed at levels of 0%, 1%, 2% and 4%. Results showed that dietary addition of blueberry powder did not affect body weight gain, feed consumption and feed conversion ratio (*p* > 0.05). No significant difference was observed between hot and cold carcass weights and carcass yield among carcass parameters (*p* > 0.05). However, significant differences were found among the blueberry-supplemented groups in terms of thigh, back and neck ratios (*p* < 0.05). In this study, it was determined that thiobarbituric acid (TBA), pH and peroxide values in breast meat samples kept at +4 °C for 1, 3, 5 and 7 days were lower in the blueberry-supplemented groups compared to the control group and these values decreased linearly as the supplement level increased (*p* < 0.05). The addition of blueberries to the quail diets resulted in similar L, a and b values in breast and thigh meat and skin among the groups (*p* > 0.05) except for the b value in thigh meat (*p* < 0.05). The findings obtained in this study revealed that although adding blueberries to the quail diet did not have a significant effect on performance, the antioxidant activity and phenolic substance content of the plant had a significant effect on increasing the shelf life of meat. It was concluded that blueberry could be used as a natural additive that may replace synthetic antioxidants.

## Simple Summary

Blueberry powder (BBP) is rich in antioxidants and minerals and has the potential to be beneficial for poultry diets. The aim of this study was to investigate the effects of adding 0, 1, 2 and 4% BBP to quail diets on growth performance, carcass traits and meat quality of quail. The results showed that BBP improved meat quality by reducing oxidative stress markers such as pH, peroxide and TBA levels in breast meat, but did not affect growth performance. The findings of this study highlight the potential of BBP as a functional feed additive to improve chicken meat quality.

## 1. Introduction

The complete ban of antibiotic growth promoters in animal feed has been in effect since 21 January 2006. This ban is based on the fact that antibiotics, which are used to promote growth and prevent infection in chickens, are detrimental to the health of both animals and humans. Following the prohibition, the poultry sector saw an up-surge in research into growth factors and natural feed additives. The constant pursuit of antibiotic alternatives in response to the growing demand for healthier animal products has led to their increased usage in animal feed [1].

It is observed that natural products and medicinal plants, which generally have positive effects on health preservation, are increasingly preferred due to their accessibility and economic reasons, whether consciously or unconsciously [2]. Poultry meat is widely recognized as a primary source of animal protein, a vital component for sustaining a nutritious diet. Poultry meat is seen as highly advantageous today due to its adequate vitamins, minerals, low fat content, easy digestibility, shorter production time compared to red meat and lower cost [3]. With the increase in income levels in many developed countries, both producers and consumers are increasingly aware of the composition and nutritional value of meat, which is a valuable food in human nutrition. This awareness is also increasing the demand for quality meat. In addition, many studies focus on improving pre-slaughter and post-slaughter quality in poultry meat. In the production of quality poultry meat, in addition to meeting the nutritional requirements of animals in an adequate and balanced manner, natural and reliable additives are added to the ration to improve the quality of the meat [4,5,6].

In recent years, especially due to the problems caused by the intensive use of anti-biotics, there has been a trend towards the use of natural and organic products in animal feeding that do not have harmful effects on both animal health and humans. In parallel with this trend, new approaches emphasizing the use of natural and reliable alternative feed additives have begun to be implemented [7]. Alternative additives commonly used in the nutrition of poultry include phytogenics, organic acids, prebiotics, probiotics, enzymes and their derivatives [8]. These feed additives are considered powerful tools for improving the health status of animals and achieving economic production. Plant extracts, which do not have harmful effects on animal health and demonstrate positive effects, have recently started to be used widely in animal feeding [9,10]. Medicinal and aromatic plants synthesize various bioactive secondary metabolites such as alkaloids, essential oils, terpenes, flavonoids, phenols, resins, saponins, etc. [11]. Aromatic plants and especially their oils are used as antimicrobial preservatives in many industries such as medicine, food, and cosmetics [12]. Their antioxidant properties, which have the ability to neutralize the effects of free radicals, have also been demonstrated in many studies [4,5,6]. Growing concerns about health hazards associated with synthetic antioxidants have led to increased efforts to identify natural antioxidant alternatives [13]. Nutritionists have begun to conduct studies to evaluate phytochemicals with antimicrobial, antiviral, antiparasitic, antifungal, antioxidant and anthelmintic properties as possible sources in order to find alternatives to antibiotics [14]. Phytochemicals found in plant products have been shown to have beneficial effects and medicinal properties with antimicrobial, antioxidant, anti-inflammatory and immunomodulatory activities without adversely affecting growth and feed utilization. Therefore, they are used as natural feed additives that promote growth in animals [15,16].

One of the products that can be used in research on the investigation of healthy and natural products in poultry feeding is blueberry (BB). Blueberries are a rich source of bioactive compounds such as phenolics, anthocyanins, carotenoids, alkaloids and vitamins, which contribute to their strong antioxidant properties [17,18,19,20,21]. Key phenolic compounds include chlorogenic acid, quercetin, kaempferol and resveratrol, which together with anthocyanins, play a major role in providing health benefits by neutralizing free radicals and reducing oxidative stress [19,20]. Okan et al. [19] determined that the total phenolic content in the fruits was 77.26–215.12 mg GAE/100 g, the total flavonoid content was 30.44–91.69 mg QE/100 g, the total anthocyanin content was 43.03–295.06 mg c3 GE/100 g and the antioxidant activities of the fruits were between DPPH 1.10–5.65 mg/mL. Phytochemicals found in the structure of blueberries include important vitamins, minerals, fatty acids and dietary fibers. Therefore, blueberries are an important source of minerals, provitamin A, B-complex vitamins and vitamin C [22]. Beyond antioxidant effects, blueberries exhibit anti-inflammatory, anticancer, and neuroprotective properties both in vitro and in vivo [23,24,25]. Blueberries have anthocyanins, and with their strong antioxidant properties, neutralize free radicals and counteract oxidative stress and reduce the occurrence of chronic diseases such as cardiovascular disease, cancer and neurological disorders [25].

The addition of BB in poultry diets has been shown to enhance the growth performance, immune function and meat quality of broilers and laying hens in previous studies [21,26,27]. Relatively limited research has been conducted on the impact of this on Japanese quails, particularly in terms of meat quality during storage. The majority of the current research focused on in vivo responses while ignoring the effects of such dietary changes on the stability and shelf life of meat after slaughter. This study aimed to address the research gap by examining the impact of BBP inclusion on the fattening performance and carcass traits of Japanese quails, as well as its effects on oxidative stability, pH parameters and physicochemical changes in breast meat stored under refrigeration. We hypothesized that the dietary addition of BBP would enhance performance and meat quality, as well as improve cold storage stability by reducing lipid oxidation, microbial load and pH variations over time. In poultry systems, this integrated approach offers new insights into the use of functional additives obtained from fruits to increase production efficiency and preserve meat.

## 2. Materials and Methods

### 2.1. Ethical Approval

The animal study protocol was approved by the Animal Research Ethics Committee of Niğde Ömer Halisdemir University (Date: 12 January 2023, approval number: 2023/03).

### 2.2. Animal, Diet and Management

A total of 480 1-day-old quail chicks were procured from the quail unit of Ayhan Şahenk Agricultural Research Center, Niğde Ömer Halisdemir University, Niğde Türkiye. The birds were weighed using an electronic scale with ±0.01 g precision, placed in 5-tier brooders and transferred to fattening cages at the end of the 2nd week. In the brooders, the temperature was set to 32–33 °C during the first week using a thermostat-controlled heater, and then the temperature was gradually reduced by 2–3 °C each week until it stabilized at 24–25 °C. The room temperature in the quail rearing room was also controlled by an air conditioner. BBP was prepared in-lab by drying whole blueberries at 50 °C and grinding them into a fine powder using a laboratory mill as the supplemented material. In the 5-week study, the quails were given commercial broiler chick starter feed containing 23% crude protein and 3100 kcal/kg metabolic energy during the experiment. All birds were fed an iso-caloric and iso-nitrogenous broiler chick starter feed for 5 wks, as per NRC [28] guidelines (Table 1).

In the study, 4 different groups were created by adding 0%, 1%, 2% and 4% blueberry powder to commercial feed. The inclusion of 4% BBP may have affected the nutrient density of the diet, potentially altering the energy–protein balance and introducing bioactive compounds that influence metabolism. Inclusion levels of BBP were selected based on previous research [26,29] and preliminary trials to assess palatability and tolerance in quails. In total, 480 1-day-old quail chicks with an average body weight of 9.73 ± 0.03 g were randomly allocated to four treatment groups, with each group consisting of four replicates of thirty chicks in each. Birds were housed at a stocking density of approximately 200 cm^2^ per bird. The trial with the quail was conducted over a period of 35 days, during which feed and water were provided ad libitum and a combination of natural and artificial lighting was supplied continuously for 24 h.

### 2.3. Blueberry Extraction Process

The BBP used in the study was prepared from commercially sourced *Vaccinium corymbosum* (highbush blueberry) obtained from Niğde, Türkiye. Ethanolic extraction of BBP was performed using a modified method based on previous literature [30,31]. To obtain blueberry extract, the material was washed and dried in an oven at 50 °C for 24 h. For the extraction process, 10 g of blueberry were ground and dissolved in 100 mL of 70% ethanol in a dark, shaking water bath set to 25 °C on an orbital shaker (500 RPM, Boeco, OS20, Hamburg, Germany). The mixture was shaken in closed glass flasks for 15 h, and at the end of this period, ultrasonic treatment was applied for 15 min in an ultrasonic water bath. The blueberry extract, dissolved in ethanol, was filtered through coarse filter paper and transferred to a rotary evaporator. The ethanol in the extracts was evaporated at 50 °C to obtain blueberry extracts. The resulting blueberry extract was stored at −80 °C for the purpose of determining the total phenolic content and antioxidant activity [26]. Prior research has demonstrated that ethanol extracts of *V. corymbosum* are abundant in anthocyanins (e.g., delphinidin, malvidin, cyanidin), flavonols (e.g., quercetin, myricetin), phenolic acids (e.g., chlorogenic acid) and other polyphenols, all of which contribute to their potent antioxidant properties [30,31].

### 2.4. Determination of Total Phenolic Content in Blueberries

To determine the total phenolic content in the blueberry extract, 100 µL of the diluted blueberry extract solution was mixed with 900 µL of distilled water, 5 mL of 0.2 N Folin–Ciocalteu reagent, and 4 mL of a saturated sodium carbonate (Na_2_CO_3_) solution (7.5 g/L). The mixture was then allowed to stand in the dark at room temperature for two hours. Afterward, the absorbance was measured at 765 nm using a spectrophotometer. The concentration of total phenolic compounds was determined using an equation derived from a standard gallic acid graph and was expressed as micrograms of gallic acid equivalent per gram (mg gallic acid/g). The results were evaluated using the previously established gallic acid curve and were reported as mg gallic acid per gram [32].

### 2.5. Determination of Antioxidant Activity in Blueberries

To determine the antioxidant activity in blueberry extract, a 7 mM ABTS solution containing 2.45 mM potassium persulfate was prepared and allowed to stand at room temperature in the dark for 12–16 h to generate the radical solution (ABTS•^+^). A series of concentrations of both the blueberry extract and Trolox were prepared to determine the antioxidant activity of the extract as Trolox equivalents. To measure this, 10 µL of the sample was added to 1 mL of ABTS•^+^, and the decrease in absorbance was monitored over 6 min. The slopes of the graphs showing percentage inhibition against concentrations were calculated. The antioxidant activity of the blueberry extract was determined as equivalent to 1 mM Trolox by comparing the slope of the blueberry extract to the slope of the Trolox concentrations. Each concentration was measured in triplicate, and all spectrophotometric readings were performed at 30 °C using microcuvettes [33].

(Example slope/slope of trolox) × dilution factor = TEAC value µM trolox where TEAC stands for Trolox Equivalent Antioxidant Capacity.

### 2.6. Performance Measurement and Carcass Characteristics Assessment

Feed intake was measured weekly on a per-replicate basis, considering the total feed consumed by the quails in each replicate. Weekly individual feed consumption was determined by dividing by the number of quails in each repetition. Throughout the study, quails’ body weight were measured individually with an electronic scale with ±0.01 g precision. Weekly body weight gains of quails were determined by subtracting the body weight of the previous week from the average body weight of each replicate each week. The feed conversion ratio (FCR) was determined by dividing the amount of feed consumed by the quails by the body weight gain.

### 2.7. Determination of Carcass Characteristics

At the end of the study, the quails were weighed to determine their body weights, and based on the average body weight of each group, 3 female and 3 male quails from each replication were slaughtered to determine carcass characteristics. After plucking the feathers of the slaughtered quails, their internal organs were removed, and their feet were cut off to determine the hot carcass weight. The carcasses were then stored at +4 °C for 24 h, and the cold carcass weight was recorded after this period. The cold carcasses were subsequently cut according to TSE (Turkish Standards Institution) standards, and the weights of the main carcass parts were determined. Additionally, the weights of the internal organs were measured and their ratios to the carcass were calculated. After determining the carcass characteristics of the slaughtered quails, 8 breast meat samples from each group were collected and stored at +4 °C for 1, 3, 5 and 7 days.

### 2.8. Peroxide Value Analysis of Breast Meat PV

Breast meat samples were collected from each group (4 males and 4 females) at each storage time point (1, 3, 5 and 7 days), that had been stored at +4 °C. To determine the oxidation state of the meat, peroxide value analysis was performed according to the AOAC 965.33 method AOAC [30]. For this purpose, the breast meat was blended and homogenized, then subjected to an extraction process to obtain the fat. From the extracted fat, 1 mL was taken and placed in 250 mL Erlenmeyer flasks. To this, 30 mL of a chloroform-acetic acid solution (3 parts chloroform and 2 parts acetic acid) was added. Subsequently, 1 mL of saturated potassium iodide (KI) solution was added, and after thorough mixing, the mixture was allowed to stand in the dark for 5 min. Afterward, 30 mL of distilled water and 4 drops of starch solution were added, and the mixture was titrated with sodium thiosulfate (Na_2_S_2_O_3_) solution. The results were calculated and expressed as peroxide value in m.equiv kg^−1^.

### 2.9. Thiobarbituric Acid (TBA) Number of Quail Breast Meat

To determine the level of lipid oxidation, thiobarbituric acid (TBA) number analysis was performed on breast meat samples from each group (4 male and 4 female) after 1, 3, 5 and 7 days of storage at +4 °C. For this purpose, fat was extracted from the breast meat samples, and 0.100 g of fat was taken and transferred into a 25 mL round-bottom flask. TBA solution was added to this fat sample, and the volume was completed to 25 mL. The mixture was then homogenized using an ultra torr mixer to achieve a homogeneous state. The homogeneous mixture was poured into a beaker, and 5 mL was transferred into tubes. To each tube, 5 mL of thiobarbituric acid was added and mixed again. The resulting mixtures in the tubes were placed in a boiling water bath at 95 °C for 2 h. After the incubation period, the samples were read in a spectrophotometer at a wavelength of 530 nm [34].

### 2.10. Determination of pH in Quail Breast Meat

At the end of the study, breast meat samples from each group (4 male and 4 female) were collected, and the pH levels of the breast meat were measured on days 1, 3, 5 and 7. A Testo 205 brand meat and food pH meter was used to determine the pH level on the day of slaughter (day 0). For this purpose, measurements were taken from three different areas of the breast meat, and the average of these values was calculated. To determine the pH value after storage at +4 °C for 1, 3, 5 and 7 days, the breast meat was blended, and a 5 g sample was mixed with distilled water to create a homogeneous mixture. The resulting homogeneous mixture was filtered, and the pH level of the breast meat was measured using a portable pH meter [35].

### 2.11. Measurement of Color Values in Quail Drumsticks and Breast Meat

The lightness (L), redness (a) and yellowness (b) values in 8 drumsticks and breast meat samples from each group were measured using a chromometer (Konica Minolta (CR-300 colorimeter). Calibration was performed with black and white caps before starting the measurements. The color of the meat was determined using the obtained L, a and b values.

### 2.12. Statistical Analysis of Data

The data were analyzed using a completely randomized design including four treatments, and statistical assessments were performed utilizing the Statistical Package for the Social Sciences (SPSS) version 18.0 (SPSS Inc., Chicago, IL, USA). Analysis of variance (ANOVA) was used to evaluate group differences, while Duncan’s multiple range test was utilized to determine significant differences where appropriate [36]. Statistical significance was determined at (*p* < 0.05), with notable group differences indicated by distinctive superscripts (a, b).

## 3. Results

### 3.1. Total Phenolic Content and Antioxidant Capacity of Blueberry Extract

In this study, blueberry extraction was performed using a solvent ratio of 70% ethanol and 30% water, and the total phenolic content in the extract obtained from the extraction process was determined. The total phenolic content of the blueberry extract was found to be 395.69 mg gallic acid equivalent (GAE) per gram. The antioxidant capacity of the blueberry extract was found to be 105.09 µmol Trolox/g.

### 3.2. The Effect of Blueberries on Performance Parameters

The body weight data of the quails fed with feed supplemented with different levels of BBP (0, 1, 2 and 4%) are presented in Table 2. It was observed that there was no significant difference in the average weekly body weight among the experimental groups in all weeks of the study (*p* > 0.05). Similarly, no significant differences were found between the males and females of all experimental groups from the 2nd week onwards (*p* > 0.05).

### 3.3. The Effects of Blueberry on Weekly Body Weight Gain, Feed Consumption, and Feed Conversion Ratio

The findings indicated that the inclusion of BBP in quail diets did not lead to statistically significant variation in body weight gain among the groups during any of the evaluated time periods (*p* > 0.05). The feed intake of all groups during the different experimental periods was similar and there were no significant differences between groups (*p* > 0.05).

Additionally, there was no statistically significant variation in the feed conversion ratio, a critical indicator of feed efficacy, among the groups that were supplemented with varying levels of BBP across all time frames (*p* > 0.05). These findings indicate that the growth performance, feed intake and conversion ratio of quails were not adversely or positively affected by the inclusion of BBP at the tested levels under the conditions of this study are presented in Table 3.

### 3.4. Effect of Blueberries on Carcass Values

At the end of the trial, which took place on day 35 (age), the average slaughter weights of the quails did not exhibit any significant differences between the groups (*p* > 0.05). Table 4 shows that the dietary addition of BBP had no significant (*p* > 0.05) effect on slaughter body weight, hot and cold carcass weight, or carcass yield in the overall quail population and in males (*p* > 0.05). However, a significant difference was observed in the slaughter body weight of female quails (*p* = 0.009), with the highest weight recorded in the 2% blueberry group. On the other hand, the groups that received 0% and 1% observed the lowest weights. Although the differences were not statistically significant, the data suggest a potential trend toward improved growth in female quails with certain levels of BBP inclusion, which may warrant further investigation.

There were no statistically significant differences (*p* > 0.05) in the hot carcass weight, cold carcass weight, or carcass yield across the dietary groups with respect to carcass traits. Additionally, when comparing the averages of males and females within the groups, there were no statistically significant differences seen in these parameters (*p* > 0.05).

### 3.5. Effect of Blueberries on Carcass Part Ratios

The proportions of the primary carcass components of the quails at the conclusion of the experiment are represented in Table 5, which demonstrates significant differences in specific areas. It was noted that there were statistically significant differences between the groups in terms of the proportions of the thighs, back and neck (*p* < 0.05). A significantly lower (*p* < 0.05) thigh weight was observed in the 1% BBP-fed female quails compared to those in the 0% and 2% BBP groups, which did not differ significantly from each other. This suggests that the inclusion of 2% BBP may have had a favorable effect on thigh development in female quails.

The addition of blueberry powder did not have an effect on the breast and wing proportions of the carcass, as shown by the fact that there were no significant differences found between the groups in terms of breast and wing proportions (*p* > 0.05). On the other hand, it was determined that back ratios showed significant differences in both genders and their overall averages (*p* < 0.05). These results emphasize the fact that the dietary inclusion of blueberry powder may not have an equal effect on all parts of the carcass.

### 3.6. Edible Organ Proportions and Abdominal Fat and pH Values

Table 6 summarizes the results of this study, which evaluated the edible internal organ ratios (heart and liver), abdominal fat ratios and pH values of quails that were fed diets supplemented with variable levels of BBP (0, 1, 2 and 4%). In the study, it was found that there were no statistically (*p* > 0.05) significant differences in these parameters between the groups. This was the case when the overall group averages as well as the averages within the male and female subgroups were taken into consideration (*p* > 0.05). The results indicate that dietary inclusion with blueberries, irrespective of dosage, does not significantly influence the proportions of heart, liver, gizzard weight, abdominal fat or pH levels in quails.

### 3.7. Determination of pH Values of Breast Meat

Following storage at a temperature of +4 degrees Celsius for 1, 3, 5 and 7 days, breast meat samples were obtained from two female and two male carcasses from each group of quails at the end of the research for the purpose of conducting a pH analysis. When compared to the control group, the groups that were supplemented with BBP consistently had lower pH values throughout all measurement days (*p* < 0.05). The pH value decreased linearly with increasing BBP inclusion in quail feeds (*p* < 0.05). These findings are reported in Table 6, which highlights the findings. Table 6 shows that pH values of quail breast meat increased significantly with storage time across all groups (*p* < 0.001). However, groups supplemented with BBP had consistently lower pH values than the control group on all storage days (*p* < 0.05), and pH decreased as the blueberry level increased.

In addition, a distinct pattern was noticed: the pH values gradually reduced as the amount of blueberry addition rose as compared to the previous readings. On the seventh day of storage, the pH values for the groups that had been supplemented with 0, 1, 2 and 4% BBP were reported as 8.08, 7.09, 6.84 and 6.41, respectively. On day 7, the pH was highest in the control group (8.08) and lowest in the 4% BBP group (6.41). BBP addition has a dose-dependent impact on reducing pH levels during storage, as shown by this result. These results indicate that the dietary addition of BBP can effectively reduce pH increase during storage, likely due to its antioxidant properties.

The lower part of Table 7 (Interaction of Storage Time over groups) further confirms that pH values significantly increased over time in all groups, with the highest values observed on day 7 (*p* < 0.001). Despite this increase, the BBP-supplemented groups maintained lower pH levels compared to the control at each time point. Significant interactions between group and storage time (*p* < 0.001) indicate that the effect of BBP on pH becomes more pronounced as storage time increases. This highlights the potential of dietary inclusion of BBP to slow pH rise and preserve meat quality during cold storage.

### 3.8. Thiobarbituric Acid (TBA) Value of the Breast Meat

As shown in Table 8, the TBA (thiobarbituric acid) values of breast meat were determined after it had been stored at a temperature of +4 degrees Celsius for 1, 3, 5 and 7 days, consecutively. Throughout all storage periods, it was noted that there were significant differences between the groups (*p* < 0.05). TBA values, which indicate lipid oxidation, significantly increased with storage time in all groups (*p* < 0.001). However, TBA values were consistently lower in the BBP-supplemented groups compared to the control. The group that was supplemented with 4% BBP displayed the lowest values (*p* < 0.05), whereas the group that was the control continuously displayed the highest TBA values, which is suggestive of higher lipid oxidation. In addition, higher amounts of inclusion of blueberry powder were shown to be correlated with a reduction in TBA values throughout all measurement days of the study. Group means ranged from 0.394 mg MDA/kg in the control to 0.129 mg MDA/kg in the 4% BBP group, demonstrating a clear dose-dependent antioxidant effect of BBP.

In the lower part of Table 8 (Interaction of Storage Time over groups), storage time means also revealed that lipid oxidation increased over time (*p* < 0.05), but to a much lesser extent in the BBP-supplemented groups. Significant group × storage time interactions (*p* < 0.001) confirm that the protective effect of BBP became more pronounced with longer storage. The findings of this study indicate that the BBP antioxidant qualities significantly reduced lipid oxidation in quail breast flesh during refrigeration, hence emphasizing the potential of the powder as a natural preservative in poultry products.

### 3.9. The Peroxide Value of Breast Meat

The breast meat samples were stored at +4 °C for 1, 3, 5 and 7 days to assess variations in peroxide values. With an increase in storage time, it was noted that there were significant changes in the peroxide values across all groups (*p* < 0.05). Table 9 shows that peroxide values, which reflect the level of primary lipid oxidation, significantly increased with storage time in all treatment groups (*p* < 0.001). However, quails fed BBP-supplemented diets had significantly lower peroxide values compared to the control group at every time point. The 4% BBP group consistently showed the lowest values, while the control group (0% BBP) had the highest, indicating a strong antioxidant effect of BBP that intensified with higher inclusion levels. Additionally, peroxide values decreased at each storage time as the concentration of BBP increased. Following a period of seven days, the peroxide values for the groups containing 0%, 1%, 2% and 4% BBP were 9.080, 8.006, 6.030 and 4.086, respectively (*p* < 0.05).

In the lower part of the table (Interaction of Storage Time over groups), the storage time means confirm a significant rise in peroxide values over time (*p* < 0.001), with values increasing from day 1 to day 7 in all groups. Significant interactions between group and storage time (*p* < 0.001) further demonstrate that the protective effect of BBP became more pronounced with extended storage. Overall, these findings highlight the effectiveness of dietary addition of BBP in reducing lipid oxidation and improving the oxidative stability of quail breast meat during cold storage.

### 3.10. Effects of Blueberry on the Color of the Thigh Meat, Thigh Skin, Breast Meat and Breast Skin

At the end of the experiment, the L (lightness), a (redness) and b (yellowness) values of the breast and thigh meat and skin were analyzed in the quails that had been slaughtered before. Table 10 shows the effect of different levels of dietary inclusion of BBP on the color values (L*, a*, b*) of quail breast and thigh meat and skin. In breast meat, a significant difference was found in L* (lightness) values (*p* = 0.049), with the highest lightness in the control group (61.92) and the lowest in the 4% BBP group (59.99). No significant differences were found in a* (redness) or b* (yellowness) values for breast meat and skin (*p* > 0.05).

For thigh meat, the dietary inclusion of BBP had a significant effect on b* (yellowness) values (*p* = 0.001), with the highest yellowness observed in the 2% BBP group (9.33), suggesting a noticeable enhancement in color. However, no significant differences were detected in L* or a* values for thigh meat, or in any color parameters for thigh skin (*p* > 0.05). These results suggest that BBP can influence certain color characteristics, particularly lightness in breast meat and yellowness in thigh meat, without affecting overall redness or skin color.

## 4. Discussion

The addition of BBP into the diet of Japanese quails significantly influenced meat quality, oxidative stability and color attributes, while it had no significant effect on performance characteristics. The results showed that dietary addition of BBP into the quail’s diet did not significantly affect performance measures, including bodyweight gain (BWG), feed intake (FI) and feed conversion ratio (FCR), which aligns with previous findings in poultry studies [29,37,38] where fruit by-products did not negatively impact growth performance despite their fiber and polyphenol content. The results indicated that the FCR, FI and BWG were not significantly influenced by the dietary inclusion of BBP at 1%, 2% or 4%. These results align with the findings reported by Hu et al. [39], who observed no significant differences in growth performance when broiler diets were supplemented with 3% blueberry or pineapple pomace. Kithama et al. [40] also noted that the addition of 0.5% and 1% low-bush blueberry pomace, regardless of enzyme supplementation, did not significantly impact overall weight growth or feed efficiency in broilers. The findings showed that the increase in the dietary addition of BBP in quail diets had no negative effect on performance. These results indicate that the fibrous nature of fruit pomace does not have a detrimental impact on nutrient utilization when it is included in poultry diets at moderate levels.

A study conducted to determine the antioxidant activity of blueberries found that the antioxidant capacity of blueberry extract was reported to be 44.6 µmol trolox/g [24,41,42]. A study found that the highest total antioxidant content value in different BB vinegar samples was 141.14 ± 6.6 μg TE/mL. It was determined that blueberry vinegar, being richer in carotenoids, phytosterols and bioactive compounds, had higher total antioxidant and total phenolic values [43]. Blueberries contain a high amount of phenolic compounds, which is why they have high antioxidant activity. The increased value of blueberries and the transition to their cultivation, making them more accessible, have further increased interest in this fruit [44]. In their study to determine the chemical composition and antioxidant properties of blueberries, they identified the physical and chemical characteristics of the fruit, noting that the water content of the fruit is 86.03% and the sugar content is 8.382 g per 100 g. They also reported the total phenolic content, tannins, flavonoids and anthocyanins as 985.17, 736.75, 84 and 99.78 mg GAE per 100 g, respectively [45]. The utilization of blueberry pomace may contribute to an improvement in the oxidative status of poultry [27]. The study by Qin et al. [26] corroborates with our findings by illustrating that the antioxidant capacity, metabolic activity and overall product quality of poultry can be improved through the dietary addition of blueberry pomace. This finding is consistent with the observed improvements in meat quality and oxidative stability in our research. Previous study has shown that dietary inclusion with grape pomace and xylo-oligosaccharides may improve the performance of broiler chickens. This highlights the potential of functional feed additives in the production of poultry that is more environmentally friendly [45,46,47]. The results of the study by Kithama et al. [40] are consistent with our findings because they highlight the impact that berry pomace addition has on the metabolism and performance of poultry. The research by Hu et al. [39] corroborates our results by showing that dietary incorporation with blueberry pomace enhances meat quality measures without adversely affecting growth performance, hence reinforcing the efficacy of blueberry-based feed additives in chicken production.

A non-significant increase in slaughter weight was observed among female quails in the 2% BBP group, despite the fact that there was no influence on the overall performance of the quails. This is partially corroborated by the results of Qin et al. [37], who observed that the dietary addition of fermented blueberry pomace (FBP) during the late laying period resulted in an increase in body weight and reproductive hormone levels in laying hens. In our study, the differential response between male and female birds may be indicative of differences in nutrient partitioning and metabolic responses, which may be influenced by sex hormones. Colombino et al. [48] observed that the incorporation of fruit pomace might improve metabolic efficiency in some bird species, particularly under oxidative or environmental stress situations.

While overall carcass characteristics were not significantly affected by the dietary inclusion of BBP, female quails in the 1% BBP group exhibited significantly lower slaughter weights, whereas the 2% and 4% BBP groups showed values comparable to the control, indicating no consistent dose-dependent effect, suggesting a potential sex-specific growth response to dietary antioxidants. The parameters of the carcass, such as hot and cold carcass weight and total yield, were not significantly affected by BBP treatment. An increase in the back ratio was observed, whereas changes in other carcass traits were either statistically insignificant or decreased. These findings suggest that the dietary addition of BBP may influence specific components of carcass composition without significantly affecting the total carcass weight. These findings are in line with the results reported by researchers [49,50,51]. In contrast, we observed significant differences in the relative proportions of the neck, back and thighs. These results corroborate those of Turcu et al. [52], who indicated that grape pomace extract impacted muscle growth and carcass composition in broilers, likely attributable to its bioactive components influencing muscle metabolism. Our findings suggest that while BBP does not enhance the total carcass output, it may have a subtle impact on muscle distribution, particularly in areas such as the thighs.

The reduced pH levels in BBP-supplemented groups likely led to a decrease in microbial development. In addition, our research on pH trends over storage time demonstrated that the dietary inclusion of blueberry powder effectively delayed the pH increase in breast meat during cold storage. Meat quality indicators were markedly improved by the dietary addition of BBP. Breast meat from BBP-fed groups exhibited significantly lower pH values during cold storage, with the lowest pH consistently observed in the 4% BBP group. Lower pH values in meat are typically associated with delayed microbial spoilage and improved shelf life. This conclusion aligns with the findings of Mauro et al. [53], who reported that berry-derived polyphenols exhibited antibacterial properties and facilitated meat preservation by sustaining reduced pH levels post slaughter. Our results’ findings are in line with the observations reported by Asghar et al. [54].

One of the most significant results of this investigation was the enhancement of the meat’s oxidative stability. The antioxidant capabilities of blueberries, abundant in polyphenols and flavonoids, resulted in decreased thiobarbituric acid (TBA) readings, signifying less lipid peroxidation and improved oxidative stability. This indicates that BBP may efficiently impede oxidative reactions, maintaining meat freshness throughout storage. It is known that the richness of poultry meat in polyunsaturated fatty acids increases its sensitivity to lipid oxidation [54]. Both thiobarbituric acid reactive substances (TBA) and peroxide levels, indicators of lipid oxidation, were considerably reduced in a dose-dependent manner in the BBP-supplemented groups. The findings corroborate the antioxidant function of blueberry polyphenols, particularly anthocyanins, which are well recognized for their lipid protective properties. The plasma lipid oxidation markers of broilers that were fed blueberry pomace were also reduced, as reported by Kithama et al. [40]. In addition, Qin et al. [37] identified elevated antioxidant enzyme activity (e.g., CAT and GSH-PX) in hens that were fed fermented blueberry pomace, which further supports the function of BB in improving antioxidant defenses. Our analysis revealed that the 4% BBP group had the lowest TBA and peroxide values, indicating its efficacy in inhibiting oxidative deterioration during cold storage. Correspondingly, both TBA and peroxide values’ key indicators of lipid oxidation were significantly reduced in BBP-supplemented groups, particularly at higher inclusion levels and longer storage durations. These findings strongly support the antioxidant potential of BBP in maintaining meat freshness and quality over time. In another study, they investigated the effect of natural antioxidant supplementation in quail, measuring the TBA values of thigh and breast meat samples on days 0 and 9 to determine lipid peroxidation. They observed that the TBA values in the groups with natural antioxidant supplementation were lower than those in the control group on both days 0 and 9 [55].

When it came to meat quality, the quails that were fed with BBP showed lower pH values in their breast meat when compared to the control group. Additionally, the pH decreased as the quantity of BBP rose. This is consistent with other research indicating that antioxidant-rich fruits, such as blueberries, enhance meat quality by sustaining lower pH levels, hence improving texture and extending shelf life. In another study, the use of BBP at 100 and 400 mg/kg as a feed additive were examined in the diets of 63-day-old chickens. They measured the pH value of the chicken breast meat and found that the pH value in the control group was higher compared to the groups with additives [55]. The addition of black cumin powder in quail feed was investigated and it was observed that the groups with additives (1%, 2% and 4%) had lower pH values compared to the control group [5]. The use of quinoa seed extract as an antioxidant source yielded similar results. The meat was stored at +4 °C in the refrigerator throughout the study, and it was reported that the pH value of the breast meat in the groups given quinoa seed extract was lower compared to the control group [4].

To determine the meat quality, they stored breast muscle pieces at 4 °C. They conducted experiments on breast meat quality and examined the color values (L*, a* and b*). They reported that the use of blueberries significantly affected the quality and color of poultry breast meat [40]. The findings of this research indicate that dietary BBP, particularly at concentrations of 2% to 4%, may significantly improve the oxidative stability and some physical quality attributes of quail meat without adversely affecting growth performance. These results align with other research [56,57,58,59,60,61] indicating the advantages of fruit pomaces, like those derived from grape, cranberry or fermented blueberry residues, in enhancing antioxidant status, meat quality and metabolic health in poultry. Despite the fact that growth performance remains largely unaffected, the use of BB as a natural antioxidant feed additive has been evidenced by improvements in meat oxidative stability, color quality and post-mortem pH control. These advantages are particularly pertinent in poultry systems that are antibiotic-free or organic, as there is a high demand for natural preservation strategies. Consequently, BBP serves as a potential natural ingredient for enhancing meat quality and extending shelf life in quail production systems. Although the findings indicate that BBP may possess antioxidant properties in quail diets, this investigation did not contain a synthetic antioxidant as a positive control. Consequently, it is impossible to draw any definitive conclusions regarding its capacity to replace commercial antioxidants. Such comparisons should be incorporated into future studies to assess the relative efficacy.

Additionally, the dietary addition of blueberry powder significantly affected meat color, particularly in the lightness (L*) and yellowness (b*) values of breast and thigh meat. A significant reduction in L* was observed in breast meat, and an increase in b* was noted in thigh meat, particularly in the 2% BBP group. These changes may be attributed to the natural pigments and phenolic compounds in blueberries, which can deposit in tissues and affect meat color, a key quality trait for consumer acceptance. This is due to the natural pigments in blueberries, particularly anthocyanins, which are renowned for enhancing meat color. Darker meat coloration is often linked to enhanced consumer perceptions of freshness and quality, especially in chicken, and may suggest increased antioxidant absorption from the diet. These pigments have the potential to bind to muscle tissues and modify their visual properties, as previously observed by Giannenas et al. [56]. They reported that the consumption of herbal and berry extracts by hens resulted in modifications to the color parameters of the meat. Although these alterations do not impact nutritional content, they may affect customer perception and acceptability. In another study, the dietary inclusion of blueberries in the diets of chickens was investigated and it was found that it had a significant effect on the lightness and yellowness of the meat. Additionally, they observed a significant increase in redness values (a*) in the groups with the additives [38]. The color, appearance, quality and freshness of meat are among the most important factors for consumers. A study was conducted to investigate the color, texture and antioxidant-related properties of meat in chickens and it was reported that blueberries had a significant effect on the lightness and yellowness of the meat [39]. This study used *Vaccinium corymbosum* (highbush blueberry), whereas others have used *Vaccinium angustifolium* (lowbush blueberry). Although both are called blueberries, *V. angustifolium* generally has higher polyphenol and anthocyanin levels, resulting in greater antioxidant capacity [41,62].

Overall, the addition of BBP to the diet of Japanese quails improved the quality of their meat by reducing pH, enhancing oxidative stability and changing the composition of their carcasses. The impact on performance varied according to the level of dietary addition. Additional study is required to investigate the long-term impacts of BBP on quail health and performance, along with the possibility of other antioxidant-rich additions to provide comparable enhancements.

## 5. Conclusions

This study showed that BBP, a rich source of phenolic compounds and antioxidants, can be added to quail diets without any negative impact on body weight gain, feed intake or feed conversion ratio. The use of blueberries significantly improved meat quality during storage in quails. The incorporation of blueberries with a concentration rate of 4% reduced the pH, peroxide and TBA values of quail breast meat, indicating improved oxidative stability and prolonged shelf life.

Its practical applicability from an economic perspective is restricted by the absence of significant enhancements in production efficiency, despite these quality-related benefits. The cost and availability of BBP may impose additional restrictions on its widespread use in commercial poultry operations. According to these results, blueberries can be included in quail diets as a natural antioxidant that will improve meat quality during storage. The comparative efficacy of BBP is restricted by the absence of a synthetic antioxidant control, despite the fact that it enhanced oxidative stability. Further study is recommended to fully understand the benefits of blueberries as a viable alternative to commercial antioxidants and their wider impacts, especially on gut microbiota in poultry production systems.

## Figures and Tables

**Table 1:** Composition and calculated nutrient content of the basal diet fed to Japanese quails throughout the experimental period.

Raw Materials	Percentage (%)	Calculated Nutrients	Value (%) or (kcal/kg)
Corn	43.30	ME (kcal/kg)	3100
Soybean meal	38.07	Crude protein	23.00
Wheat bran	12.00	Dry matter	89.35
Soybean oil	3.50	Crude fat	6.77
CaCO_3_	0.82	Crude ash	5.53
DCP	0.78	Crude fiber	5.38
Salt	0.30	Lysine	1.45
Lysine	0.52	Methionine	0.67
Methionine	0.39	Methionine + Cystine	1.07
Threonine	0.12	Calcium	0.91
Vitamin mix *	0.10	Total phosphorus	0.45
Mineral mix **	0.10		

DCP: Dicalcium phosphate; * Vitamin-mineral premix per kilogram of feed includes 12,000 IU vitamin A, 5000 IU vitamin D3, 50 mg vitamin E, 10 mg vitamin K3, 5 mg vitamin B2, 20 mg vitamin B12, 6 mg vitamin B1, 5 mg vitamin B6, 50 mg niacin, 25 mg folic acid, 30 mg biotin, 75 mg pantothenic acid, 175 mg choline chloride; ** Mineral mix per kilogram of feed contains 100 mg manganese, 80 mg iron, 60 mg zinc, 150 mg cobalt, 12 mg copper, 200 mg selenium.

**Table 2:** Effect of dietary addition of blueberry powder on weekly body weight of experimental groups (g).

Days		Groups				SEM	*p*-Value
		0% BBP	1% BBP	2% BBP	4% BBP		
1.		9.70	9.70	9.71	9.81	0.035	0.647
7.		36.79	36.27	35.53	35.73	0.203	0.121
14.		95.26	94.15	93.93	92.90	0.466	0.355
21.	M	163.13	163.87	163.84	164.21	1.051	0.985
	F	170.29	165.40	165.85	164.76	1.046	0.246
	Mean	166.74	164.66	165.07	164.47	0.745	0.694
28.	M	224.28	226.46	227.16	225.71	1.227	0.871
	F	234.90	230.41	234.51	232.96	1.190	0.552
	Mean	229.82	228.32	231.48	228.94	0.875	0.611
35.	M	266.29	270.62	270.71	272.18	1.655	0.618
	F	295.86	288.97	295.10	294.31	1.700	0.504
	Mean	281.56	279.15	285.13	281.87	1.341	0.473

SEM: Standard Error of the Mean; *p*: Significance Level; 0% BBP: 0% blueberry powder; 1% BBP: 1% blueberry powder; 2% BBP: 2% blueberry powder; 4% BBP: 4% blueberry powder; M: male; F: female.

**Table 3:** Effect of dietary addition of blueberry powder on weekly body weight gain (g), feed intake (g) and feed conversion ratio.

Days	Groups				SEM	*p*-Value
	0% BBP	1% BBP	2% BBP	4% BBP		
Body Weight Gain (g)						
1–14 days	85.56	84.41	84.22	83.08	0.905	0.847
15–35 days	186.22	184.68	191.24	188.73	1.120	0.173
1–35 days	271.78	269.10	275.43	271.81	1.384	0.487
Feed Intake (g/bird)						
1–14 days	153.11	152.08	149.89	153.33	1.651	0.901
15–35 days	631.50	636.30	630.90	632.00	3.171	0.944
1–35 days	784.60	788.38	780.79	785.33	3.692	0.929
Feed Conversion Ratio (g feed intake/g body weight gain)						
1–14 days	1.79	1.80	1.78	1.85	0.016	0.511
15–35 days	3.39	3.45	3.30	3.35	0.028	0.346
1–35 days	2.88	2.93	2.83	2.89	0.020	0.457

SEM: Standard Error of the Mean; *p*: Significance Level; 0% BBP: 0% blueberry powder; 1% BBP: 1% blueberry powder; 2% BBP: 2% blueberry powder; 4% BBP: 4% blueberry powder.

**Table 4:** Effect of dietary addition of blueberry powder on slaughter body weight (g), hot and cold carcass weight (g) and carcass yield (%) of experimental groups.

Groups	Slaughter Body Weight (g)	Hot Carcass Weight (g)	Cold Carcass Weight (g)	Carcass Yield (%)
Total Birds (Mean)				
0% BBP	281.87	211.30	210.34	74.67
1% BBP	278.53	209.79	207.79	74.62
2% BBP	282.48	213.34	209.27	74.13
4% BBP	283.83	212.26	208.74	73.58
SEM	1.227	0.872	0.877	0.236
*p*-Value	0.477	0.530	0.782	0.330
Males				
0% BBP	271.77	207.45	206.80	76.08
1% BBP	274.63	209.90	207.35	75.51
2% BBP	271.04	207.20	203.47	75.09
4% BBP	275.31	208.98	204.96	74.44
SEM	1.058	0.925	0.984	0.244
*p*-Value	0.409	0.710	0.500	0.106
Females				
0% BBP	291.95 ^a^	215.13	213.88	73.26
1% BBP	282.43 ^b^	209.68	208.23	73.73
2% BBP	293.92 ^a^	219.49	215.07	73.19
4% BBP	292.33 ^a^	215.54	212.52	72.72
SEM	1.381	1.326	1.285	0.347
*p*-Value	0.009	0.069	0.261	0.801

a,b means different letters in the same column are significantly different from each other (*p* < 0.05). SEM: Standard Error of the Mean; 0% BBP: 0% blueberry powder; 1% BBP: 1% blueberry powder; 2% BBP: 2% blueberry powder; 4% BBP: 4% blueberry powder.

**Table 5:** Effect of different levels of dietary addition of blueberry powder on the ratio of carcass parts of the quail (%).

Groups	Thighs	Breast	Wings	Back	Neck
Total Birds (Mean)					
0% BBP	33.67 ^a^	36.15	9.22	12.83 ^b^	6.09 ^a^
1%BBP	33.00 ^b^	36.39	9.35	13.23 ^ab^	5.30 ^b^
2% BBP	33.81 ^a^	35.18	9.31	13.70 ^a^	5.63 ^ab^
4% BBP	33.55 ^ab^	36.37	9.42	12.91 ^b^	5.19 ^b^
SEM	0.111	0.183	0.057	0.101	0.092
*p*-Value	0.005	0.059	0.670	0.009	0.002
Males					
0% BBP	33.63	35.95	9.17	12.85 ^b^	6.14
1%BBP	33.37	35.67	9.22	13.86 ^a^	5.41
2% BBP	33.90	34.64	9.21	13.74 ^a^	5.76
4% BBP	33.91	36.00	9.30	12.71 ^b^	5.58
SEM	0.147	0.248	0.090	0.150	0.115
*p*-Value	0.524	0.178	0.966	0.006	0.143
Females					
0% BBP	33.70 ^a^	36.35	9.27	12.81 ^b^	6.04 ^a^
1%BBP	32.64 ^b^	37.11	9.49	12.60 ^b^	5.19 ^b^
2% BBP	33.73 ^a^	35.72	9.42	13.65 ^a^	5.49 ^ab^
4% BBP	33.19 ^ab^	36.73	9.54	13.12 ^ab^	4.80 ^b^
SEM	0.163	0.254	0.068	0.135	0.141
*p*-Value	0.050	0.259	0.553	0.030	0.011

Means without a common superscript in a column are significantly different (*p* < 0.05). M: Male; F: Female; SEM: Standard Error of the Mean; 0% BBP: 0% blueberry powder; 1% BBP: 1% blueberry powder; 2% BBP: 2% blueberry powder; 4% BBP: 4% blueberry powder.

**Table 6:** Effect of different levels of dietary addition of blueberry powder on edible organs, abdominal fat (%) and pH value.

Groups	Heart	Liver	Gizzard	Abdominal Fat	pH
Total Birds (Mean)					
0% BBP	1.33	3.42	2.87	2.12	5.75
1%BBP	1.32	3.30	2.90	2.00	5.69
2% BBP	1.31	3.32	2.85	2.18	5.68
4% BBP	1.27	3.60	2.93	1.88	5.72
SEM	0.018	0.081	0.038	0.077	0.020
*p*-Value	0.651	0.571	0.873	0.517	0.658
Males					
0% BBP	1.31	2.79	2.89	2.27	5.78
1%BBP	1.33	3.02	2.67	2.06	5.69
2% BBP	1.31	3.09	2.72	2.38	5.71
4% BBP	1.27	3.32	2.86	1.92	5.81
SEM	0.025	0.079	0.048	0.120	0.025
*p*-Value	0.910	0.119	0.284	0.544	0.327
Females					
0% BBP	1.36	4.04	2.84	1.98	5.71
1%BBP	1.33	3.59	3.14	1.95	5.69
2% BBP	1.32	3.54	2.99	1.99	5.66
4% BBP	1.27	3.87	3.00	1.84	5.64
SEM	0.026	0.123	0.055	0.095	0.030
*p*-Value	0.722	0.439	0.292	0.947	0.819

M: Male; F: Female; SEM: Standard Error of the Mean; 0% BBP: 0% blueberry powder; 1% BBP: 1% blueberry powder; 2% BBP: 2% blueberry powder; 4% BBP: 4% blueberry powder.

**Table 7:** Effect of varying levels of dietary addition of blueberry powder and storage time on pH levels in breast meat of quails.

Groups	Storage Time (Days)				
	1	3	5	7	Group Means
0% BBP	6.25 ^a^	6.45 ^a^	7.14 ^a^	8.08 ^a^	6.98 ^a^
1% BBP	6.24 ^a^	6.34 ^b^	6.86 ^b^	7.09 ^b^	6.63 ^ab^
2% BBP	6.15 ^b^	6.24 ^c^	6.57 ^c^	6.84 ^c^	6.45 ^bc^
4% BBP	6.07 ^c^	6.13 ^d^	6.24 ^d^	6.41 ^d^	6.22 ^c^
SEM	0.023	0.035	0.102	0.185	0.074
*p*-Value	0.000	0.000	0.000	0.000	0.001
Linear	0.000	0.000	0.000	0.000	0.000
Quadratic	0.105	0.918	0.918	0.700	0.666
Cubic	0.203	0.890	0.890	0.905	0.041
**Storage Time (Days)**	**Groups**				
	**0% BBP**	**1% BBP**	**2% BBP**	**4% BBP**	**Storage Time Means**
1	6.25 ^d^	6.24 ^c^	6.15 ^d^	6.07 ^c^	6.18 ^c^
3	6.45 ^c^	6.34 ^c^	6.24 ^c^	6.13 ^bc^	6.29 ^c^
5	7.14 ^b^	6.86 ^b^	6.57 ^b^	6.24 ^b^	6.70 ^b^
7	8.08 ^a^	7.09 ^a^	6.84 ^a^	6.41 ^a^	7.10 ^a^
SEM	0.215	0.108	0.083	0.041	0.074
*p*-Value	0.000	0.000	0.000	0.001	0.000
Linear	0.000	0.000	0.000	0.000	0.000
Quadratic	0.000	0.267	0.001	0.195	0.187
Cubic	0.031	0.024	0.004	0.967	0.525
**Interactions**					
**Groups**		**Storage Time**		**Group × Storage Time**	
0.000		0.000		0.000	

Means without a common superscript in a column are significantly different (*p* < 0.05). 0% BBP: 0% blueberry powder; 1% BBP: 1% blueberry powder; 2% BBP: 2% blueberry powder; 4% BBP: 4% blueberry powder.

**Table 8:** Effect of varying levels of dietary addition of blueberry powder and storage time on thiobarbituric acid (TBA) value (mg MDA/kg) in breast meat of quails.

Groups	Storage Time (Days)				
	1	3	5	7	Group Means
0% BBP	0.199 ^a^	0.349 ^a^	0.495 ^a^	0.532 ^a^	0.394 ^a^
1% BBP	0.190 ^b^	0.304 ^b^	0.326 ^b^	0.314 ^b^	0.283 ^b^
2% BBP	0.178 ^c^	0.260 ^c^	0.263 ^c^	0.243 ^c^	0.236 ^b^
4% BBP	0.117 ^d^	0.129 ^d^	0.145 ^d^	0.125 ^d^	0.129 ^c^
SEM	0.009	0.025	0.038	0.044	0.017
*p*-Value	0.000	0.000	0.000	0.000	0.000
Linear	0.000	0.000	0.000	0.000	0.000
Quadratic	0.000	0.004	0.105	0.000	0.930
Cubic	0.001	0.100	0.035	0.000	0.231
**Storage Time (Days)**	**Groups**				
	**0% BBP**	**1% BBP**	**2% BBP**	**4% BBP**	**Storage Time Means**
1	0.199 ^c^	0.190 ^b^	0.178 ^b^	0.117 ^b^	0.171 ^b^
3	0.349 ^b^	0.304 ^a^	0.260 ^a^	0.129 ^ab^	0.260 ^ab^
5	0.495 ^a^	0.326 ^a^	0.263 ^a^	0.145 ^a^	0.307 ^a^
7	0.532 ^a^	0.314 ^a^	0.243 ^a^	0.125 ^b^	0.303 ^a^
SEM	0.040	0.017	0.011	0.004	0.018
*p*-Value	0.000	0.000	0.001	0.024	0.017
Linear	0.000	0.000	0.003	0.126	0.004
Quadratic	0.001	0.000	0.001	0.012	0.159
Cubic	0.088	0.251	0.235	0.119	0.965
**Interactions**					
**Groups**		**Storage Time**		**Group × Storage Time**	
0.000		0.000		0.000	

Means without a common superscript in a column are significantly different (*p* < 0.05). 0% BBP: 0% blueberry powder; 1% BBP: 1% blueberry powder; 2% BBP: 2% blueberry powder; 4% BBP: 4% blueberry powder.

**Table 9:** Effect of varying levels of dietary addition of blueberry powder and storage time on peroxide values in breast meat of quails.

Groups	Storage Time (Days)				
	1	3	5	7	Group Means
0% BBP	4.090 ^a^	6.053 ^a^	8.020 ^a^	9.080 ^a^	6.810 ^a^
1% BBP	3.060 ^b^	4.023 ^b^	7.100 ^b^	8.006 ^b^	5.547 ^a^
2% BBP	2.130 ^c^	3.046 ^c^	5.036 ^c^	6.030 ^c^	4.060 ^b^
4% BBP	1.050 ^d^	2.046 ^d^	3.013 ^d^	4.086 ^d^	2.549 ^c^
SEM	0.340	0.446	0.583	0.576	0.340
*p*-Value	0.000	0.000	0.000	0.000	0.000
Linear	0.000	0.000	0.000	0.000	0.000
Quadratic	0.708	0.000	0.000	0.000	0.810
Cubic	0.411	0.000	0.001	0.004	0.427
**Storage Time (Days)**	**Groups**				
	**0% BBP**	**1% BBP**	**2% BBP**	**4% BBP**	**Storage Time Means**
1	4.090 ^d^	3.060 ^d^	2.130 ^d^	1.050 ^d^	2.582 ^b^
3	6.053 ^c^	4.023 ^c^	3.046 ^c^	2.046 ^c^	3.792 ^b^
5	8.020 ^b^	7.100 ^b^	5.036 ^b^	3.013 ^b^	5.792 ^a^
7	9.080 ^a^	8.006 ^a^	6.030 ^a^	4.086 ^a^	6.800 ^a^
SEM	0.576	0.621	0.467	0.340	0.340
*p*-Value	0.000	0.000	0.000	0.000	0.000
Linear	0.000	0.000	0.000	0.000	0.000
Quadratic	0.000	0.654	0.492	0.346	0.840
Cubic	0.007	0.000	0.000	0.448	0427
**Interactions**					
**Groups**		**Storage Time**		**Group × Strorage Time**	
0.000		0.000		0.000	

Means without a common superscript in a column are significantly different (*p* < 0.05). 0% BBP: 0% blueberry powder; 1% BBP: 1% blueberry powder; 2% BBP: 2% blueberry powder; 4% BBP: 4% blueberry powder.

**Table 10:** Effect of different levels of dietary addition of blueberry powder on thigh meat, thigh skin, breast meat and breast skin L, a and b values.

Groups	Breast Meat			Breast Skin		
	L*	a*	b*	L*	a*	b*
0% BBP	61.92 ± 0.631 ^a^	3.41 ± 0.272	10.03 ± 0.424	68.52 ± 1.594	2.95 ± 0.267	8.87 ± 0.513
1% BBP	60.94 ± 0.548 ^ab^	2.83 ± 0.218	9.87 ± 0.351	66.95 ± 0.483	2.65 ± 0.229	8.20 ± 0.513
2% BBP	60.20 ± 0.374 ^b^	3.38 ± 0.219	10.32 ± 0.256	68.35 ± 0.672	2.71 ± 0.199	8.99 ± 0.713
4% BBP	59.99 ± 0.533 ^b^	3.50 ± 0.329	10.25 ± 0.207	69.90 ± 0.666	3.10 ± 0.183	9.34 ± 0.506
SEM	0.272	0.132	0.159	0.483	0.110	0.283
*p*-Value	0.049	0.272	0.749	0.198	0.447	0.552
	**Thigh Meat**			**Thigh Skin**		
	L*	a*	b*	L*	a*	b*
0% BBP	56.09 ± 0.465	3.34 ± 0.188	5.96 ± 0.345 ^b^	60.96 ± 1.057	1.63 ± 0.120	6.01 ± 0.601
1% BBP	55.97 ± 0.435	2.92 ± 0.198	6.65 ± 0.408 ^b^	62.00 ± 0.755	2.24 ± 0.330	6.89 ± 0.681
2% BBP	57.16 ± 0.530	3.43 ± 0.157	9.33 ± 0.598 ^a^	61.33 ± 0.547	2.18 ± 0.144	5.45 ± 0.399
4% BBP	55.31 ± 0.492	3.20 ± 0.183	6.11 ± 0.384 ^b^	60.89 ± 0.906	2.08 ± 0.196	6.59 ± 0.690
SEM	0.246	0.092	0.259	0.414	0.108	0.302
*p*-Value	0.063	0.221	0.001	0.777	0.180	0.352

Means without a common superscript in a column are significantly different (*p* < 0.05). SEM: Standard Error of the Mean; *p*: Significance Level; BBP: Blueberry Powder; L*: Relative Lightness; a*: Relative Redness; b*: Relative Yellowness; 0% BBP: 0% blueberry powder; 1% BBP: 1% blueberry powder; 2% BBP: 2% blueberry powder; 4% BBP: 4% blueberry powder.

## Data Availability

The data presented in this study are available on request from the corresponding author.
